# Structural elements´ availability for the provision of Prevention of Mother-to-Child Transmission of HIV services among health facilities in the Volta Region of Ghana

**DOI:** 10.11604/pamj.2022.41.87.29458

**Published:** 2022-02-01

**Authors:** Christine Edem Dzamboe, Emmanuel Manu, Fortress Yayra Aku, Elvis Enowbeyang Tarkang

**Affiliations:** 1 Department of Population and Behavioural Science, School of Public Health, University of Health and Allied Sciences PMB 31, Ho, Volta Region, Ghana,; 2 Department of Epidemiology and Biostatistics, School of Public Health, University of Health and Allied Sciences PMB 31, Ho, Ghana,; 3 HIV/AIDS Prevention Research Network Cameroon, PO Box 36, Kumba, Cameroon

**Keywords:** Structural elements, health facilities, Volta Region, Ghana

## Abstract

**Introduction:**

despite advancement in global efforts to prevent mother-to-child transmission (PMTCT) of HIV, more work needs to be done to achieve the desired results in most African countries including Ghana. Inadequate structural elements can hinder the progress made so far in PMTCT of HIV. This study assessed the availability of structural elements for the provision of PMTCT of HIV services among health facilities in the Volta region of Ghana.

**Methods:**

a descriptive cross-sectional design was used among thirty-two health facilities. Data obtained were analysed using Stata version 14.0 and the Chi-square test was used to determine associations at the 0.05 level of significance.

**Results:**

a majority of the health facilities were Health Centers (50.0%) and most (43.8%) were located in rural areas. Only 9.5% of health practitioners at the Community Health Planning and Services (CHPS) Compounds and Mission-based Hospitals were trained in PMTCT, while 50.5% and 30.5% of health practitioners providing services at the Health Centers and District Hospitals respectively were trained in PMTCT. About 40.0% of District Hospitals had one room with auditory and visual privacy for PMTCT services. While all Mission-based and District Hospitals had ART regimens, no CHPS compound had, and only 8 (50.0%) of the Health Centers had ART regimens.

**Conclusion:**

there is a need for regular training of the health care practitioners providing PMTCT services. Also, programme managers should invest in PMTCT commodities, especially ART regimens, at the lower levels of healthcare for a holistic PMTCT service provision.

## Introduction

Globally, there have been many efforts to end the Acquired Immune Deficiency Syndrome (AIDS) pandemic by the year 2030 [1]. As part of the measures to reverse the devastating effects of the Human Immunodeficiency Virus (HIV), prevention remains a key strategy. To ensure this, global efforts to prevent mother-to-child transmission (PMTCT) of HIV have been integrated. Mother-to-child transmission (MTCT) of HIV can occur during pregnancy, delivery or through breastfeeding and causes high morbidity and mortality among children under five years [2]. Reducing the rate of MTCT of HIV could be achieved through routine HIV testing and counselling (HTC) of all pregnant women, provision of antiretroviral therapy (ART), safe delivery practices, and counselling and support on infant feeding. Worldwide, there is an estimated shortage of about 2.4 million Doctors, Nurses and Midwives in handling matters related to HIV counselling and service provision [3]. Results from a study in South Africa revealed that the national in-utero MTCT of HIV rate was 0.9%, while the provincial level ranged from 0.6% to 1.3% and the district level from 0.4% to 1.9% [4]. A similar study in South Africa found the in-utero transmission of HIV to be less than 1% [5]. Efforts have been made in Ghana to scale up access to ART especially among pregnant women to reduce MTCT of HIV [6]. However, a study reported the MTCT of HIV rate to be 2.3%, which is deemed high [2]. Among the factors that have been observed to present challenges in early infant diagnosis of HIV are lack of effective diagnostic tools and laboratory support, late return of results, late or non-reporting of mothers, loss to follow up, lack of husband support, independent maternal income source, as well challenges with the disclosure of HIV status [2].

Between 2015 and 2017, the Volta Regional Health Directorate (VRHD) reported an increase in ANC clients tested and receiving post-test counselling from 77.1 to 95.2 percent, and an increase in HIV-positive cases from 1.1 to 1.4 percent. The number of HIV-exposed infants also increased from 313 to 488 in the region [7]. These results could be attributed to the efficacy of structural elements in the provision of PMTC of HIV services, among other factors. Structural elements of care also referred to as inputs of care, comprise all the inputs that enhance a health facility´s readiness to provide the intended services when clients come for the required services. Structural elements are the characteristics of the setting in which healthcare takes place. Structural elements for rendering PMTCT of HIV services at health facilities include trained human resources, physical infrastructure (space), supplies (HIV test kits for adults and infants, ART regimens for adults and infants, Information Educational and Communication [IEC] material, and Co-trimoxazole) and equipment [8]. The structural barriers to PMTCT of HIV as reported in Accra, Ghana [9] include inadequate counselors, inadequate working space, limited laboratory capacity for testing and lack of means of transport for monitoring and evaluation activities. The expectation from the full implementation of the revised Ghana national PMTCT of HIV guidelines in 2014 at all levels of healthcare delivery was that it will lead to the attainment of the national goal of virtual elimination of Mother-to-Child transmission (e-MTCT) of HIV by 2015 [3]. This expectation has not been met, even though the median HIV prevalence rate for pregnant women has declined to 2.1% in 2017 [7]. Until more studies are conducted to ascertain the specific structural element challenges in different regions and health facilities of Ghana, appropriate remedies cannot be implemented to reduce the rate of PMTCT of HIV. It is against this background that the current study seeks to assess the structural elements´ availability for the provision of PMTCT of HIV services across health facilities in the Volta region of Ghana.

## Methods

**Study site:** the Volta region is one of the sixteen regions of Ghana and is located in the eastern part of the country. It shares boundaries in the north with the Oti region, to the south with the Gulf of Guinea, in the west with the Volta Lake and to the east with the Republic of Togo. The region is divided into 18 administrative districts. Based on the 2010 national population and housing census, the projected population of the region for 2016 was 2,456,520 with an annual growth rate of 4.0% (before the Oti region was carved out of it). The region has a total of 684 health facilities, which comprise 441 Community-based Health Planning and Services (CHPS) compounds, 44 clinics, 29 hospitals, 153 health centers, 14 maternity homes, 4 polyclinics and 1 regional hospital [10].

**Study population:** the study population consisted of key informants of health facilities providing PMTCT of HIV services in the Volta Region of Ghana.

**Inclusion and exclusion criteria:** the study included only key informants of PMTCT of HIV-providing health facilities who consented to participate in the study. Key informants who were on leave were excluded from the study.

**Study design:** a descriptive cross-sectional design, using a quantitative data collection method, was used. This design was employed as it captures data at a single point in time and relatively inexpensive and can be used within a short duration [11].

**Sample size determination:** all the 32 health facilities providing PMTCT of HIV services in the Volta region were included in the study.

**Sampling method and data collection tool:** the purposive sampling technique was used to select all the 32 PMTCT of HIV-providing facilities in the Volta region of Ghana. One key informant was selected from each facility to be part of the study. The personnel in charge of PMTCT of HIV services were purposively selected to give responses regarding the availability of structural elements for the provision of PMTCT of HIV services at their respective facilities. In the case where there were two or more persons in charge, a simple random sampling was used to select one as the key informant if they were all the same ranks. However, the senior-most person in charge was selected in a case where the persons in charge were of various ranks in the unit. An assessment tool developed by the family health international (FHI) institute for HIV/AIDS for assessing PMTCT of HIV services was adopted and used to assess the structural elements (human resources, infrastructure, equipment and supplies) [12]. Information on human resources (numbers of providers, types of providers, qualifications, supervision, and training), infrastructure (space) and supplies (HIV test kits for adults and infants, ART regimens for adults and infants, IEC material, and Co-trimoxazole) for PMTCT of HIV was obtained from the selected key informants of the health facilities.

**Data collection procedure:** data collection took place at the health facilities where the key informants who were eligible were interviewed. Five research assistants were trained on how to collect data using the data collection tool. The questionnaire was pre-tested in the Ho Municipal Hospital in the Volta region. The date and time for data collection at the health facilities were communicated to the key informants at the various facilities before data collection. Data were collected using face-to-face interviews.

**Data analysis:** data were entered into Epi-Data 3.1 software, cleaned and validated to ensure quality before analysis began. The cleaned database was exported to Stata version 14.0 for analysis. Frequencies and percentages were used to describe the data. Chi-square test of association was used to determine the association between the structural elements and the health facility types and location.

**Ethical issues:** ethical approval for the study was obtained from the University of Health and Allied Sciences (UHAS) Research Ethics Committee (UHAS-REC A.7[4]18-19) before the commencement of the study. Before inclusion into the study, written informed consent was obtained from each key informant. Permission was sought from the Volta regional health directorate and the selected facilities. The facilities were given special identification codes without using their names. This was to ensure anonymity and confidentiality.

## Results

**General characteristics of the health facilities providing PMTCT of HIV services:** half of the health facilities providing PMTCT of HIV services were health centers 16(50.0%) and most of the facilities 14(43.8%) were located in rural areas (Table 1).

**Table 1:** general characteristics of the health facilities providing PMTCT services

Variable	Frequency (N=32)	Percent (%)
**Type of facility**		
Community-based Health Planning and Services (CHPS)	7	21.9
Health Centers	16	50.0
Mission-based Hospital	4	12.5
District Hospital	5	15.6
**Location of facility**		
Rural	14	43.8
Semi-Urban	8	25.0
Urban	10	31.2

### Human resources available for the provision of PMTCT of HIV services

Of the 32 health facilities, a total of 197 health care professionals provided PMTCT of HIV services, a majority of whom were Midwives 57 (28.9%). However, only 103 (52.3%) were trained in PMTCT of HIV service delivery, and mostly Midwives 41 (39.8%) (Table 2). On human resources availability by facility, 5 (14.3%) Physician Assistants were present at the CHPS compounds and none was trained on the delivery of PMTCT of HIV, while none were present at the District Hospitals. Five (16.1%) and 4 (44.5%) Midwives were present and trained respectively at the Mission-based Hospitals while 17(35.5%) Midwives were present at the District Hospitals of which 14(48.3%) were trained. The number of Nurses that were present and trained at the CHPS compounds was 13(37.1) and 3(33%) respectively; 6 (12.5%) were present at the District Hospitals and 2(6.9%) were trained (Table 3). No doctor was present at the CHPS compounds and the Health Centers; however, at the District Hospitals, 5 (10.4%) were present, of which 2 (6.9%) were trained. Likewise, at the CHPS compounds and the Health Centers, no laboratory staff was present. Five (10.4%) laboratory staff were present at the District Hospitals and 2 (6.9%) were trained. Regarding Counselors, 11 (31.4%) were present at the CHPS compounds, followed by 16 (19.3%) at the Health Centers. There were no data Managers at the CHPS compounds; however, 5 (10.4%) were present at the District Hospitals, of which 4 (13.8%) were trained (Table 3).

**Table 2:** human resources availability

Service provider	Providers of PMTCT services n (%)	Providers trained in PMTCT services n (%)
Physician Assistants	18 (9.1)	7 (6.8)
Midwives	57 (28.9)	41 (39.8)
Nurses	54 (27.4)	17 (16.5)
Doctor (s)	8 (4.1)	2 (1.9)
Laboratory staff	9 (4.6)	9 (8.8)
Counsellors	32 (16.2)	20 (19.4)
Pharmacy staff (s)	6 (3.1)	2 (1.9)
Data Manager (s)	13 (6.6)	5 (4.9)
**Total**	**197 (100.0)**	**103 (52.3)**

**Table 3:** human resources availability by health facility

	CHPS		Health Center		M-Hospital		D-Hospital	
Service provider	Present n (%)	Trained n (%)	Present n (%)	Trained n (%)	Present n (%)	Trained n (%)	Present n (%)	Trained n (%)
**Physician Assistants**	5 (14.3)	0 (0.0)	5 (6.0)	5 (10.4)	8 (25.8)	2 (22.2)	0 (0.0)	0 (0.0)
**Midwives**	6 (17.1)	4 (44.5)	29 (34.9)	19 (39.6)	5 (16.1)	4 (44.5)	17 (35.5)	14 (48.3)
**Nurses**	13 (37.1)	3 (33.3)	29 (34.9)	10 (20.8)	6 (19.4)	2 (22.2)	6 (12.5)	2 (6.9)
**Doctor(s)**	0 (0.0)	0 (0.0)	0 (0.0)	0 (0.0)	3 (9.7)	0 (0.0)	5 (10.4)	2 (6. 9)
**Laboratory staff**	0 (0.0)	0 (0.0)	0 (0.0)	0 (0.0)	4 (12.9)	1 (11.1)	5 (10.4)	2 (6.9)
**Counselors**	11 (31.4)	2 (22.2)	16 (19.3)	12 (25.0)	0 (0.0)	0 (0.0)	5 (10.4)	4 (13.8)
**Pharmacy staff (s)**	0 (0.0)	0 (0.0)	1 (1.2)	1 (2.1)	0 (0.0)	0 (0.0)	5 (10.4)	1 (3.4)
**Data Manager (s)**	0 (0.0)	0 (0.0)	3 (3.6)	1 (2.1)	5 (16.1)	0 (0.0)	5 (10.4)	4 (13.8)
Total	**35 (17.8)**	**9 (9.5)**	**83 (42.1)**	**48 (50.5)**	**31 (15.7)**	**9 (9.5)**	**48 (24.4)**	**29 (30.5)**

(CHPS=Community-Based Health Planning and Services, H/C= Health Center, M-Hospital= Mission-Based Hospital, D-Hospital= District Hospital)

### Counselling room available for PMTCT of HIV services

Considering the availability of rooms for providing PMTCT of HIV services, 6(85.7%) CHPS compounds and 2(40.0%) District Hospitals had one room. The type and the location of the facilities were not significantly associated with the number of rooms used for providing PMTCT of HIV services (Table 4). Analysing the auditory privacy in the health facilities in terms of availability, all the Mission-based and District Hospitals had auditory privacy. There was no significant association between the type and location of facilities and the availability of auditory privacy. The availability of visual privacy was also analysed and revealed that 6(85.7%) CHPS compounds and 4 (100.0%) Mission-based Hospitals had visual privacy. There was no significant association between the type and location of facilities and the availability of visual privacy (Table 4).

**Table 4:** counselling room availability

	Type of facility					Location			
Variable	CHPS	H/C	M-Hospital	D-Hospital	χ2 (p-value)	Rural	Semi-Urban	Urban	χ2 (p-value)
**Number of rooms used for HIV/PMTCT related issues**									
One	6 (85.7)	12 (75.0)	3 (75.0)	2 (40.0)		10 (71.4)	8 (100.0)	5 (50.0)	
Two	0 (0.0)	4 (25.0)	1 (25.0)	2 (40.0)		3 (21.4)	0 (0.0)	4 (40.0)	
Three	1 (14.3)	0 (0.0)	0 (0.0)	1 (20.0)	6.78 (0.342)	1 (7.1)	0 (0.0)	1 (10.0)	5.54 (0.236)
**Auditory privacy**									
Available	6 (85.7)	15 (93.7)	4 (100.0)	5 (100.0)		13 (92.9)	7 (87.5)	10 (100.0)	
Not available	1 (14.3)	1 (6.3)	0 (0.0)	0 (0.0)	1.37 (0.712)	1 (7.1)	1 (12.5)	0 (0.0)	1.22 (0.54)
**Visual privacy**									
Available	6 (85.7)	15 (93.7)	4 (100.0)	5 (100.0)		13 (92.9)	7 (87.5)	10 (100.0)	
Not available	1 (14.3)	1 (6.3)	0 (0.0)	0 (0.0)	1.37 (0.712)	1 (7.1)	1 (12.5)	0 (0.0)	1.22 (0.54)

(CHPS=Community-Based Health Planning and Services, H/C= Health Center, M-Hospital= Mission-Based Hospital, D-Hospital= District Hospital)

### Availability of supplies for infection control

As part of measures to control infections, the number of CHPS compounds that had gloves was 6(85.7%). However, all (100%) Health Centers, District and Mission-based Hospitals had gloves. All the facilities in the rural and urban areas had gloves to control infections; however, 7(87.5%) facilities in the semi-urban areas had gloves for infection control. Neither the type nor the location of the facilities was significantly associated with the availability of gloves. All the facilities in the rural, semi-urban and urban areas had sharp boxes, disposable needles and syringes and running water for controlling infections. Regarding hand-washing items, all the facilities in the rural and semi-urban areas had the items (Table 5).

**Table 5:** supplies for infection control

	Type of facility [n (%)]						Location [n (%)]			
Variable	CHPS		H/C	M-Hospital	D-Hospital	χ2 (p-value)	Rural	Semi-Urban	Urban	χ2 (p-value)
**Gloves**										
Yes	6 (85.7)		16 (100)	4 (100)	5 (100)		14 (100.0)	7 (87.5)	10 (100.0)	
No	1 (14.3)		0 (0.0)	0 (0.0)	0 (0.0)	3.69 (0.297)	0 (0.0)	1 (12.5)	0 (0.0)	3.10 (0.213)
**Sharp box**										
Yes	7 (100)	16 (100)		4 (100)	5 (100)		14 (100.0)	8 (100.0)	10 (100.0)	
No	0 (0.0)	0 (0.0)		0 (0.0)	0 (0.0)		0 (0.0)	0 (0.0)	0 (0.0)	
**Disposable needles and syringes**										
Yes	7 (100)		16 (100)	4 (100)	5 (100)		14 (100.0)	8 (100.0)	10 (100.0)	
No	0 (0.0)		0 (0.0)	0 (0.0)	0 (0.0)		0 (0.0)	0 (0.0)	0 (0.0)	
**Running water**										
Yes	7 (100)		16 (100)	4 (100)	5 (100)		14 (100.0)	8 (100.0)	10 (100.0)	
No	0 (0.0)		0 (0.0)	0 (0.0)	0 (0.0)		0 (0.0)	0 (0.0)	0 (0.0)	
**Hand washing items**										
Yes	7 (100)		15 (93.7)	4 (100)	5 (100)		14 (100.0)	8 (100.0)	9 (90.0)	
No	0 (0.0)		1 (6.3)	0 (0.0)	0 (0.0)	1.03 (0.793)	0 (0.0)	0 (0.0)	1 (10.0)	2.27 (0.321)

(CHPS=Community-Based Health Planning and Services, H/C= Health Center, M-Hospital= Mission-Based Hospital, D-Hospital= District Hospital)

### Availability of PMTCT of HIV commodities

Regarding the availability of PMTCT of HIV commodities, no CHPS compound had ART regimens available; 8(50.0%) Health Centers had ART regimens and all the Mission-based Hospitals, as well as the District Hospitals, had ART regimens. There was a (χ^2^=11.92, p-value=0.008) significant association between the type of facility and the availability of ART regimens. Based on location, 4(28.6%) facilities in the rural areas, 2(25.0) in the semi-urban areas and 9 (90.0%) in the urban areas had ART regimens. There was a (χ^2^=10.89, p-value=0.004) significant association between the location of the facility and the availability of ART regimens (Table 6). There was no ARV syrup for infants available at the CHPS compounds but all, 5(100%) of the District Hospitals had them. There was a statistically significant association between the type of facility and availability of ARV syrup for infants (χ^2^=12.31, p-value=0.006). Three (21.4%) health facilities in rural areas, 2(25.0%) in the semi-urban areas and 8(80.0%) in the urban areas had ARV syrup for infants. There was a statistically significant association between the location of the facilities and the availability of ARV syrup for infants (χ^2^=9.38, p-value=0.009) (Table 6). Concerning the availability of HIV test kits, all the health facilities despite their locations (rural, semi-urban, urban) had test kits for adults. HIV test kits for infants were available in 3 (42.9%) CHPS compounds and all the 5(100.0%) District Hospitals. There was no statistically significant association between the types of facility and the availability of HIV test kits for infants. Regarding the location of facilities with HIV test kits for infants, 4(28.6%) facilities in the rural areas, 4(50.0%) in the semi-urban areas and 9 (90.0%) in the urban areas had HIV test kits for infants. There was a statistically significant association between the location of the facilities and the availability of HIV test kits for infants (χ^2^=8.88, p-value=0.012) (Table 6). Information, education and communication (IEC) materials were available in 3(42.9%) CHPS compounds and all 4 (100.0%) the Mission-based Hospitals as well all 5(100.0%) the District Hospitals. Among the facilities in the rural areas, 10(71.4%) had IEC materials; IEC materials were available in 4(50.0%) facilities in the semi-urban areas and 9(90.0%) facilities in the urban areas. Neither the type nor the location of the facilities had a statistically significant association with the availability of IEC materials. Co-trimoxazole was available in 3(42.9%) CHPS compounds, 9(56.2%) Health Centers, 2(50.0%) Mission-based Hospitals and all the 5(100.0%) District Hospitals. There was no statistically significant association between the type and the location of the facilities and the availability of Co-trimoxazole (Table 6).

**Table 6:** availability of PMTCT commodities

	Type of facility					Location			
Variable	CHPS	H/C	M-Hospital	D-Hospital	χ2 (p-value)	Rural	Semi-Urban	Urban	χ2 (p-value)
**ART regimens**									
Available	0 (0.0)	8 (50.0)	2 (50.0)	5 (100.0)		4 (28.6)	2 (25.0)	9 (90.0)	
Not available	7 (100.0)	8 (50.0)	2 (50.0)	0 (0.0)	**11.92 (0.008)**	10 (71.4)	6 (75.0)	1 (10.0)	**10.89 (0.004)**
**ARV syrup for infants**									
Available	0 (0.0)	6 (37.5)	2 (50.0)	5 (100.0)		3 (21.4)	2 (25.0)	8 (80.0)	
Not available	7 (100.0)	10 (62.5)	2 (50.0)	0 (0.0)	**12.31 (0.006)**	11 (78.6)	6 (75.0)	2 (20.0)	**9.38 (0.009)**
**HIV test kits for adults**									
Available	7 (100.0)	16 (100.0)	4 (100.0)	5 (100.0)		14 (100.0)	8 (100.0)	10 (100.0)	
Not available	0 (0.0)	0 (0.0)	0 (0.0)	0 (0.0)		0 (0.0)	0 (0.0)	0 (0.0)	
**HIV test kits for infants**									
Available	3 (42.9)	6 (37.5)	3 (75.0)	5 (100.0)		4 (28.6)	4 (50.0)	9 (90.0)	
Not available	4 (57.1)	10 (62.5)	1 (25.0)	0 (0.0)	7.05 (0.070)	10 (71.4)	4 (50.0)	1 (10.0)	**8.88 (0.012)**
**IEC materials**									
Available	3 (42.9)	11 (68.7)	4 (100.0)	5 (100.0)		10 (71.4)	4 (50.0)	9 (90.0)	
Not available	4 (57.1)	5 (31.3)	0 (0.0)	0 (0.0)	6.52 (0.089)	4 (28.6)	4 (50.0)	1 (10.0)	3.52 (0.172)
**Co-trimoxazole**									
Available	3 (42.9)	9 (56.2)	2 (50.0)	5 (100.0		7 (50.0)	4 (50.0)	8 (80.0)	
Not available	4 (57.1)	7 (43.8)	2 (50.0)	0 (0.0)	4.42 (0.219)	7 (50.0)	4 (50.0)	2 (20.0)	2.57 (0.277)

(CHPS=Community-Based Health Planning and Services, H/C= Health Center, M-Hospital= Mission-Based Hospital, D-Hospital= District Hospital), PMTCT: Prevention of Mother-to-Child Transmission

### Frequency of running out of PMTCT of HIV commodities

From Table 7, ART regimens often ran out in all 7(100.0%) CHPS compounds, but in just 1(20.0%) District Hospital. There was a statistically significant association (?2=12.83, p-value=0.046) between the type of facility and ART regimens running out. Ten (71.4%) facilities in the rural areas, 5(62.5%) and 3(30.0%) in the semi-urban and urban areas respectively often ran out of ART regimens. The was no statistically significant association between the location of facilities and ART regimens running out. ARV syrup for infants often ran out in all 7(100.0%) CHPS compounds but in none of the District Hospitals. There was a statistically significant association (?2=19.49, p-value=0.003) between the type of facility and ARV syrup for infants running out. Based on the location of the facilities, 11(78.6%) facilities in the rural areas, 5(62.5%) in the semi-urban areas and 3(30.0%) in the urban areas often ran out of ARV syrup for infants. There was no statistically significant association between the location of the facilities and ARV syrups for infants running out.

**Table 7:** frequency of running out of PMTCT commodities

	Type of facility					Location			
Variable	CHPS	H/C	M-Hospital	D-Hospital	χ2 (p-value)	Rural	Semi-Urban	Urban	χ2 (p-value)
**ART regimens**									
Few times	0 (0.0)	0 (0.0)	0 (0.0)	1 (20.0)		0 (0.0)	0 (0.0)	1 (10.0)	
Never/Rarely	0 (0.0)	8 (50.0)	2 (50.0)	3 (60.0)		4 (28.6)	3 (37.5)	6 (60.0)	
Often	7 (100.0)	8 (50.0)	2 (50.0)	1 (20.0)	**12.83 (0.046)**	10 (71.4)	5 (62.5)	3 (30.0)	5.50 (0.240)
**ARV syrup for infants**									
Few times	0 (0.0)	0 (0.0)	0 (0.0)	2 (40.0)		0 (0.0)	0 (0.0)	2 (20.0)	
Never/Rarely	0 (0.0)	6 (37.5)	2 (50.0)	3 (60.0)		3 (21.4)	3 (37.5)	5 (50.0)	
Often	7 (100.0)	10 (62.5)	2 (50.0)	0 (0.0)	**19.49 (0.003)**	11 (78.6)	5 (62.5)	3 (30.0)	8.15 (0.086)
**HIV test kits for adults**									
Few times	1 (14.3)	4 (25.0)	0 (0.0)	2 (40.0)		3 (21.4)	2 (25.0)	2 (20.0)	
Never/Rarely	6 (85.7)	10 (62.7)	4 (100.0)	3 (60.0)		10 (71.4)	6 (75.0)	7 (70.0)	
Often	0 (0.0)	2 (12.5)	0 (0.0)	0 (0.0)	4.80 (0.569)	1 (7.2)	0 (0.0)	1 (10.0)	0.81 (0.937)
**HIV test kits for infants**									
Few times	0 (0.0)	1 (6.3)	0 (0.0)	1 (20.0)		0 (0.0)	0 (0.0)	2 (20.0)	
Never/Rarely	4 (57.1)	6 (37.5)	3 (75.0)	3 (60.0)		6 (42.9)	4 (50.0)	6 (60.0)	
Often	3 (42.9)	9 (56.2)	1 (25.0)	1 (20.0)	4.91 (0.555)	8 (57.1)	4 (50.0)	2 (20.0)	6.68 (0.154)
**IEC materials**									
Few times	0 (0.0)	2 (12.5)	0 (0.0)	2 (40.0)		0 (0.0)	1 (12.5)	3 (30.0)	
Never/Rarely	4 (57.1)	8 (50.0)	4 (100)	3 (60.0)		10 (71.4)	3 (37.5)	6 (60.0)	
Often	3 (42.9)	6 (37.5)	0 (0.0)	0 (0.0)	9.33 (0.156)	4 (28.6)	4 (50.0)	1 (10.0)	7.72 (0.102)
**Co-trimoxazole**									
Few times	0 (0.0)	2 (12.5)	0 (0.0)	3 (60.0)		0 (0.0)	1 (12.5)	4 (40.0)	
Never/Rarely	3 (42.9)	8 (50.0)	2 (50.0)	2 (40.0)		7 (50.0)	4 (50.0)	4 (50.0)	
Often	4 (57.1)	6 (37.5)	2 (50.0)	0 (0.0)	10.99 (0.088)	7 (50.0)	3 (37.5)	2 (20.0)	7.59 (0.108)

(CHPS=Community-Based Health Planning and Services, H/C= Health Center, M-Hospital= Mission-Based Hospital, D-Hospital= District Hospital)

No CHPS compound, 2(12.5%) Health Centers and no Mission-based Hospital and District Hospital often ran out of HIV test kits for adults. One (7.2%) facility in the rural areas, none in the semi-urban areas and 1(10.0%) in the urban areas often ran out of HIV test kits for adults. There was no statistically significant association between the location of the facilities and HIV test kits for adults running out. Information, Education and Communication (IEC) materials often ran out in 3(42.9%) CHPS compounds, but not in the Mission-based and District Hospitals. Of the facilities, 4(28.6%), 4(50%) and 1(10%) in the rural, the semi-urban and the urban areas respectively often ran out of IEC materials. The type of facility or their locations was not statistically significantly associated with the frequency of running out of IEC materials. Also, 4(57.1%) CHPS compounds and no District Hospital ran out of Co-trimoxazole. Concerning the location, 7(50%) facilities in the rural areas, 3(37.5%) in the semi-urban areas and 2(20%) in the urban areas often ran out of Co-trimoxazole. Neither the type of facility nor their location was statistically significantly associated with the frequency of running out of Co-trimoxazole.

## Discussion

The current cross-sectional study examined the structural elements´ availability for the provision of PMTCT of HIV services among health facilities in the Volta region of Ghana. The health workers providing PMTCT of HIV services were Midwives, Nurses, Laboratory Staff, Physician Assistants, Doctors and Pharmacists. However, Midwives and Nurses formed a greater proportion. This is consistent with the findings of studies conducted in Uganda and Malawi, which established that the health personnel available for the provision of PMTCT of HIV services to pregnant and breastfeeding women were principally trained Midwives and Nurses [13, 14]. The findings of the current study could be because in Ghana, the Ministry of Health (MOH) has positioned PMTCT of HIV services under maternal and child health services, particularly in antenatal care (ANC) and postnatal care (PNC) services, which are managed mainly by Midwives and Nurses [3]. This could explain the larger number of Midwives and Nurses in the provision of PMTCT of HIV services as compared to other cadres. With this system in place, pregnant and breastfeeding women are more likely to encounter the same service providers they met while receiving ANC and PNC services for PMTCT of HIV services, and this could contribute to the effectiveness and quality of the services provided. However, up to 47.7% of the health care professionals providing PMTCT of HIV services were not trained in PMTCT of HIV service delivery. This result is consistent with that of a cross-sectional study conducted in Tema General Hospital, Ghana, which revealed that the majority of PMTCT of HIV service providers did not have any formal training in PMTCT service delivery [15]. It also affirms the assertion of WHO that there is a shortage of trained workforce in most countries in the world in the delivery of HIV services [8]. Also, a study conducted in the Accra Metropolis of Ghana identified inadequate in-service training as a key reason for the way PMTCT of HIV services are delivered with outdated information and only few nurses had received some training in PMTCT of HIV service delivery [16]. It was further reported in the Accra metropolis of Ghana that most of the counsellors involved in HTC had not been through the foundational training organised by the Ghana Health Service (GHS), but had received on-the-job training from colleagues who had attended the GHS training sessions. Only one of the key informants had attended an initial training session organised by the GHS [17].

However, in Uganda, a descriptive cross-sectional study among 17 government health facilities in the Soroti District showed that all the health workers providing PMTCT of HIV services were trained in PMTCT of HIV service delivery [18]. The difference between the result of the current study and that of Uganda could be because the current study was conducted in five Districts whilst the Uganda study purposively used only one District, which probably might have had all its health care practitioners providing PMTCT of HIV services trained at that time. The current finding calls for the need to equip all health staff involved in PMTCT of HIV service delivery with the requisite knowledge on PMTCT of HIV service delivery. This will enable them to provide adequate services to their clients. In the current study, the majority of health facilities had only one room used for PMTCT of HIV service delivery (71.9%), which had both auditory and visual privacy. This result is consistent with that of a study conducted in Uganda, which reported that about 58.8% of the health facilities studied had only one room that was used for PMTCT of HIV-related services [16]. Although, only one room was used for PMTCT of HIV services by the majority of the facilities in the current study, auditory and visual privacy was ensured, which to a large extent could contribute to clients´ satisfaction. This could go a long way to encourage clients to cooperate with the service providers during service delivery.

Supplies for infection control such as sharp boxes, disposable needles and syringes, running water, gloves and handwashing items were available in all 32 facilities. This is in line with the WHO guidelines on the use of antiretroviral drugs for treating and preventing HIV infection, which state emphatically that routine equipment is necessary to support infection control [19]. This suggests that PMTCT of HIV service providers have access to the necessary equipment for their personal safety. The risk of contracting HIV to some extent is high if items such as test kits and syringes are handled inappropriately. It is, therefore, encouraging to provide all the needed logistics for infection control to the PMTCT of HIV-providing facilities. The availability of infection control supplies as found in the current study may reflect the relevance the various health facilities place on infection control, in providing quality PMTCT of HIV services. The wearing of disposable gloves as reported in the current study is a key component of good quality PMTCT of HIV service delivery [20]. It was also reported in the current study that most of the PMTCT of HIV-providing facilities did not have ART regimens (100% of the CHPS compounds, 50% of Health Centers, and 50% of the Mission Hospitals), and 100% of the CHPS, 50% of Health Centers, 50% of Mission Hospitals and 20% of District Hospitals did not have them quite often. In total, 46.9% of the PMTCT-providing facilities did not have ART regimens and 56.3% did not have them quite often. These findings support the assertion of the Ghana AIDS Commission, which states that the non-availability of ARVs at all PMTCT of HIV sites is a major challenge regarding the PMTCT of HIV programme [21]. The findings of the current study are similar to those of a study conducted in Ghana, which revealed that the challenges encountered by facilities include a shortage of test regimens and drugs [22]. This also corroborates with the results of a cross-sectional study in Uganda, which reported that about 30% of the health facilities did not have ARV regimens [18].

Following the Alma-Ata declaration on primary health care (PHC) of 1978 [23], Ghana implemented the CHPS in 2000 [24]. As a national health system initiative, the CHPS concept is aimed at providing accessible PHC through the reduction of physical and geographical limitations to health care accessibility in deprived communities in Ghana [25]. The role of CHPS in PHC implementation is thus to bring health services to local communities, develop sustainable volunteerism and community health action, empower vulnerable populations such as women and enhance healthcare provider-community interaction [24]. Primary healthcare, thus CHPS, is key to attaining Universal Health Coverage (UHC), which was defined as essential health care made universally accessible to individuals and acceptable to them, through their full participation and at a cost that the community could afford [23]. Therefore, the effective functioning of CHPS within rural communities is important in achieving UHC [26]. The Sustainable Development Goal (SDG) 3, which seeks to ensure healthy lives and promote wellbeing for all ages by the year 2030 [27], is one of the strategies geared towards achieving UHC, especially in low- and middle-income countries like Ghana. Therefore, the unavailability of ART regimens and ARV syrups for children in the CHPS compounds could pose serious public health challenges to quality PMTCT of HIV service delivery in rural communities. The Ghana Health Service and Managers of CHPS should always ensure the availability of PMTCT commodities to ensure quality PMTCT of HIV service delivery to the rural population.

Also, the current study revealed that 28.6%, 50.0% and 90.0% of the facilities in the rural, semi-urban and urban areas respectively had HIV test kits for infants available. There was a statistically significant association between the location of the facilities and the availability of HIV test kits for infants (χ^2^=8.88, p-value=0.012). This indicates that facilities in the rural areas could be deprived of early infant diagnosis of HIV, leaving most cases undetected. The finding is in line with a similar study conducted in Tanzania, which showed that the unavailability of test kits at health facilities was a reason for the non-uptake of the test [28]. The current findings, however, contradict Ghana´s national guidelines for PMTCT of HIV, which states that ARVs and other logistics such as test kits shall be procured solely by the Ministry of Health (MOH) in Ghana and that all facilities accredited for PMTCT of HIV shall be supplied ARVs in line with the supply chain management of MOH [3]. Although the MOH is responsible for the provision of ART regimens, there are challenges faced by PMTCT of HIV-providing facilities in accessing them both at regional and national medical stores, which is similar to all other health care commodities [3]. This suggests that attention is needed to scale up the regular and consistent provision of ART regimens and test kits for infants. Also, service providers must carefully invest in monitoring stock supply and usage of ART regimens at the facility level. Furthermore, most of the facilities in the current study had IEC materials available and only the CHPS compounds and Health Centers had their IEC materials often running out. This contradicts a similar study conducted in Accra, Ghana, which reported that the facilities lacked IEC materials for client education [15]. The results indicate that the clients visiting the facilities in the current study will be inspired and educated about prevention, care and/or treatment of HIV/AIDS and will have a better understanding of PMTCT of HIV. The current study was conducted in one region of Ghana; therefore, the results cannot be generalised to entire Ghana.

## Conclusion

This study revealed that most of the facilities providing PMTCT of HIV services were health centers and most were located in rural areas. The level of training of health care workers providing PMTCT of HIV services was inadequate in some facilities, while most of the facilities used one room with auditory and visual privacy for PMTCT of HIV service delivery. Supplies for infection control and PMTCT of HIV commodities were available in a majority of the facilities except for ART regimens, which were only available in just a few of the facilities. Also, most of the facilities often ran out of supplies of PMTCT of HIV commodities, especially those in the rural areas. Based on the results of the current study, health care practitioners providing PMTCT of HIV services should be trained regularly to equip them with the knowledge needed to execute their duties. PMTCT of HIV commodities, especially ART regimens, should be provided to the PMTCT of HIV-providing facilities regularly to provide holistic services to clients, especially in the rural areas.

### What is known about this topic

- Mother-to-child transmission (MTCT) of HIV can occur during pregnancy, delivery or through breastfeeding;
- PMTCT of HIV could be achieved through routine HTC of all pregnant women, provision of antiretroviral therapy (ART), safe delivery practices, and counselling and support on infant feeding;
- The infrastructure (space) at the facilities, supplies (HIV test kits for adults and infants, ART regimens for adults and infants, Information Educational and Communication [IEC] material, and Co-trimoxazole) and trained human resources are the structural elements that affect PMTCT of HIV service provision.

### What this study adds

- Most of the PMTCT of HIV service providers at the facilities are not trained in PMTCT of HIV service delivery;
- Most of the facilities have rooms with audio and visual privacy for the delivery of PMTCT of HIV services;
- PMTCT of HIV commodities were more available in facilities located in the urban areas and less available in the CHPS compounds.
